# Effectiveness of acupotomy combined with nerve block therapy for cervical radiculopathy: A systematic review and meta-analysis

**DOI:** 10.1097/MD.0000000000042771

**Published:** 2025-06-13

**Authors:** Hyeon-Kyu Choi, Sang-Hyun Lee, Jin-Hyun Lee, Sooil Choi, Sukhee Park, Young Su Lim, Hye-Jung Kim, Young Il Kim, Tae-Yong Park

**Affiliations:** a Department of Acupuncture and Moxibustion Medicine, Daejeon University Daejeon Korean Medicine Hospital, Daejeon, Republic of Korea; b Seonwoon Korean Medicine Hospital, Jinju, Gyeongnam, Republic of Korea; c Institute for Integrative Medicine, Catholic Kwandong University, International St. Mary’s Hospital, Incheon, Republic of Korea; d Department of Anesthesiology and Pain Medicine, Catholic Kwandong University, International St. Mary’s Hospital, Incheon, Republic of Korea; e Department of Family Medicine, Catholic Kwandong University, International St. Mary’s Hospital, Incheon, Republic of Korea.

**Keywords:** acupotomy, cervical radiculopathy, meta-analysis, nerve block, systematic review

## Abstract

**Background:**

This systematic review and meta-analysis aimed to evaluate the effectiveness and safety of combining acupotomy with nerve block therapy (NBT) for cervical radiculopathy (CR) compared to NBT alone.

**Methods:**

A comprehensive search was conducted across multiple databases to identify randomized controlled trials (RCTs) investigating the combined use of acupotomy and NBT for CR. Studies were assessed for risk of bias using the Cochrane Risk of Bias 2 tool. Data were synthesized through meta-analysis where applicable.

**Results:**

Four RCTs with a total of 540 patients were included. Meta-analysis showed that the combination of acupotomy and NBT significantly improved the total effective rate compared to NBT alone (risk ratio 1.16, 95% confidence interval 1.08–1.24, *P* < .0001). However, no significant pain reduction was observed based on the pain visual analog scale (SMD − 2.55, 95% confidence interval ‐5.32 to 0.22, *P* = .07), and there was substantial heterogeneity among the included studies (I² = 99%). The overall risk of bias was high, and safety data were limited, with only one study reporting adverse events.

**Conclusion:**

The findings suggest that acupotomy combined with NBT may enhance treatment effectiveness for CR, particularly in terms of overall therapeutic response. However, due to the high risk of bias, study heterogeneity, and insufficient safety reporting, further well-designed, large-scale RCTs with long-term follow-ups are needed to establish robust clinical evidence.

## 1. Introduction

Cervical radiculopathy (CR) results from physical and chemical disturbances affecting the cervical nerve roots, caused by soft tissue damage, cervical scoliosis or kyphosis, intervertebral disc protrusion, spinal canal stenosis, facet joint degeneration, or intervertebral foramen narrowing. Symptoms include cervical pain and radiating pain in the upper extremities, caused by mechanical compression of the nerve roots. This compression can trigger an inflammatory response, leading to paresthesia and muscle weakness in the affected areas.^[1]^ The incidence of CR is approximately 107.3 per 100,000 in men and 63.5 per 100,000 in women. CR entails significant socioeconomic costs, as it mainly affects people in their 40s and 50s.^[2]^ Since most patients respond well to conservative treatment, nonsurgical management is generally recommended unless severe conditions, such as myelopathy, are present.^[3]^

Common nonsurgical treatments for CR include immobilization, physiotherapy, traction, manual therapy, medications, and nerve block therapy (NBT).^[4]^ Among them, NBT is typically administered as a form of conservative treatment aimed at mitigating a patient’s symptoms.^[5]^ The most commonly used spinal NBTs, administered through the injection of local anesthetics or analgesics around the spine, are as follows: medial branch block, which blocks the medial branch of the facet joint; selective nerve root block (SNRB), which targets specific nerve roots; and epidural steroid injection, which delivers steroids into the epidural space.^[6–8]^ These interventions provide proven, albeit temporary, relief of neuropathic pain.^[9]^

Acupotomy, a specialized form of acupuncture, uses knife-edged needles to release adhesions, nodules, and fibrotic tissues caused by soft tissue damage, thereby alleviating pain and improving mobility.^[10]^ It has been reported to be significantly more effective than conventional or electric acupuncture in relieving pressure on blood vessels and nerves affected by soft tissue damage.^[11]^ As the procedure carries a low risk of infection, it is a preferred method for treating various musculoskeletal disorders.^[12]^ For instance, there have been reports of successful treatment of CR, including herniated intervertebral discs, with acupotomy.^[13]^

Although both NBT and acupotomy are widely used for CR, clinical evidence regarding their combined effectiveness and safety remains limited. Acupotomy mechanically releases adhesions, whereas NBT chemically suppresses inflammation. Their combination may yield clinically significant benefits, but systematic research is needed to validate its effectiveness. In this study, we review the existing literature on NBT and acupotomy according to the Preferred Reporting Items for Systematic Reviews and Meta-Analyses guidelines to analyze the possible effectiveness and safety of using both methods concurrently to treat CR.^[14]^

## 2. Methods

### 2.1. Research

The protocol of this systematic review (SR) was registered with the Prospective Register of Systematic Reviews on January 1, 2025 (CRD42024629350). Relevant studies were researched and retrieved on January 7, 2025 using electronic databases, including the Cochrane Central Register of Controlled Trials, Embase, Ovid, and PubMed, all for studies published in English. The Chinese National Knowledge Infrastructure Database was searched for studies published in Chinese. The Korea Citation Index, Korean Studies Information Service System, Oriental Medicine Advanced Searching Integrated System, Research Information Sharing Service, and ScienceOn were searched for studies published in Korean (Table S1, Supplemental Digital Content, https://links.lww.com/MD/P142).

We did not limit our search to a certain language or range of publication dates. Medical Subject Headings terms related to acupotomy and CR were incorporated into the search strategy and adapted to match the appropriate language for each database.

### 2.2. Inclusion and exclusion criteria

We formulated core questions according to the Patient Intervention Control Outcome-Study Design format to guide our selection of studies to review. The population included patients who presented with clinical CR symptoms or whose medical imaging led to diagnoses of diseases capable of inducing CR (e.g., herniated intervertebral discs). Our population was limited to adult patients aged 18 and over but did not distinguish patients in terms of sex, symptom severity, or CR duration. For intervention, we selected studies that reported the effects of combining acupotomy and NBT without discrimination based on the methods, dosages, frequencies, or durations of either treatment. For comparison, we chose studies that reported on the effects of administering NBT alone. Studies on CR treatments that did not involve NBT were excluded.

Regarding outcomes, we included all studies assessing the effects of the given treatments on reducing pain and/or improving function. We sought to measure primary outcomes in terms of cervical pain or radiating upper extremity pain, measured using either the pain visual analog scale (VAS) or the numerical rating scale. We then estimated secondary outcomes based on the neck disability index (NDI) and total effective rate (TER). Adverse events (AEs) were surveyed as potential indicators of non-safety. We confined our review to studies centered on randomized controlled trials (RCTs), excluding studies using non-randomized trials, animal samples, reporting on cellular experiments, reporting on individual cases, taking a retrospective approach, establishing protocols, or published as part of conferences.

### 2.3. Literature selection and sampling

Two independent researchers (HKC and JHL) were tasked with selecting relevant literature and sorting it using Endnote 20.0.1 (Clarivate Analytics, Boston, MA). After removing duplicate studies, the researchers sorted the remainder by subjecting their titles and abstracts to the given selection protocol. The researchers then reviewed the main texts of the chosen studies in their original languages to arrive at the final list of studies for review. When they differed on whether a given study should be included or excluded, a third person (YIK) intervened as an additional discussant in the selection process. Then, they sampled the selected literature to obtain data, such as the sizes and demographic characteristics of the study populations, administered interventions, comparisons of interventions, intervention durations, assessment tools, outcomes, and AEs. Using the Standards for Reporting Interventions in Clinical Trials of Acupuncture checklist, details on the acupotomy methods administered in the applicable studies were also obtained.^[15]^

### 2.4. Assessing the quality of the literature

Per the Preferred Reporting Items for Systematic Reviews and Meta-Analyses guidelines, we assessed the biases in the chosen studies using Cochrane Risk of Bias 2 tool.^[16]^ Specifically, we assessed the risks of bias across 3 levels (low, some concern, and high) and 5 domains (randomization processes, deviations from intended interventions, missing outcome data, outcome measurements, and selection of the reported results). Two researchers (HKC and JHL) evaluated the bias independent of one another. When the 2 disagreed, the third person (YIK) intervened as an additional discussant.

### 2.5. Method of analysis

Selected studies with the same research designs (interventions, comparisons, assessment tools, etc) were subjected to meta-analysis. For dichotomous dependent variables, we applied a risk ratio of 95% and a confidence interval (CI) of 95%. For continuous dependent variables, we subjected the final values, along with a CI of 95%, to Cochrane Review Manager (revman 5.4). We evaluated heterogeneity using Higgins’ I^2^ test. When I^2^ < 50%, we used a fixed-effect model. When I^2^ ≥ 50%, we used a random-effect model. We report only the numbers of AEs observed without treating them in the statistical analysis. To test the robustness and sensitivity of our findings, we performed a sensitivity analysis, removing study after study from highly heterogeneous groups and subjecting the rest to repeated meta-analyses.^[17]^

### 2.6. Testing for publication bias

We used a funnel plot to determine whether the studies included in our review were subject to publication bias. A symmetric graph suggests a low probability of bias, whereas asymmetry suggests a high probability.

## 3. Results

### 3.1. Selection of literature

Our searches returned 104 relevant studies from international databases, including 8 on Cochrane Central Register of Controlled Trials, 4 on Embase, 3 on Pubmed, 3 on Ovid, 85 on Chinese National Knowledge Infrastructure Database and one on Research Information Sharing Service.

After removing 14 studies that were identical and therefore redundant, we reviewed the titles and abstracts of the remaining 90 and removed 60 studies whose titles and abstracts did not follow the inclusion criteria. This left us with 30 studies.

**Figure 1.**
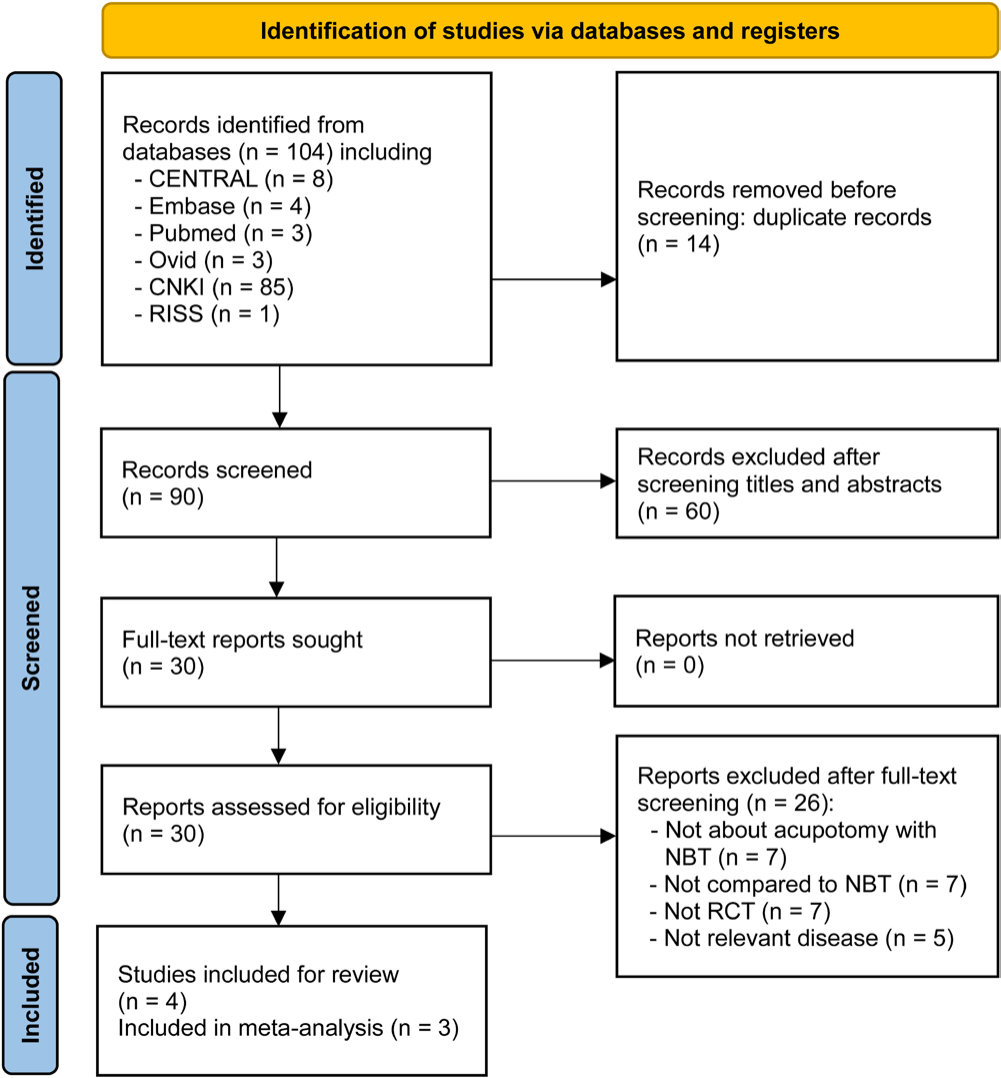
PRISMA flowchart. PRISMA = Preferred Reporting Items for Systematic Reviews and meta-analyses.

The main texts of the 30 studies were reviewed in their original languages, leading to the removal of 7 studies that involved interventions other than acupotomy and NBT, 7 with control groups that were not appropriate for our purpose, 7 that did not involve RCTs, and 5 with inappropriate disease. This left us with 4 studies to review (Fig. 1).^[18–21]^

### 3.2. Review and analysis

Four studies we reviewed were distributed between 2012 and 2023. The studies involved 540 patients in total, including 270 in treatment groups and 270 in control groups. All 3 studies that conducted follow-up observations performed observations for 3 months after their trials concluded.

Across the 4 studies, we found that 5 assessment tools were used. Pain VAS and TER emerged as the most popular, reported by 3 studies, followed by evaluation score, excellent and good rate, and NDI in one study each. Lu et al^[19]^ utilized the evaluation score, which quantifies patients’ symptoms and physical examination findings to objectively assess treatment effectiveness. This scoring system has a maximum of 35 points and includes assessments of neck and arm pain, neck tenderness, cervical range of motion, sensory dysfunction, upper limb muscle strength reduction, reflex changes, the brachial plexus traction test, and the spurling test. Meanwhile, Pu et al^[21]^ applied the excellent and good rate, which evaluates patients based on the Odom criteria clinical curative effect evaluation standard. Patients were classified into 4 grades according to their symptoms, and the proportion of those rated as excellent or good was calculated.

Three of 4 studies compared the effectiveness of administering SNRB alone versus administering SNRB with acupotomy. Only one study compared paravertebral block alone with paravertebral block and acupotomy concurrent treatment (Table 1).

**Table 1:** Characteristics of the included studies.

Author (year)	Age (mean ± SD)	Male/female	Sample size (TG:CG)	Disease type	Periods of illness	Intervention	Outcome	F/U period
Zhang (2012)^[18]^	TG: 31–68 (45.25 ± 7.15) CG: 28–69 (46.67 ± 8.19)	TG: 53/57 CG: 51/59	220 (110:110)	Cervical spondylopathy	TG: 6 months–10 years CG: 5 months–10 years	TG: Acupotomy + SNRB CG: SNRB	1. Pain VAS 2. TER (%)	N.R.
Lu (2013)^[19]^	TG: 34–56 (43.63 ± 5.55) CG: 32–58 (42.60 ± 6.58)	TG: 12/18 CG: 13/17	60 (30:30)	CSR	TG: 14.47 ± 6.10 months CG: 13.97 ± 10.24 months	TG: Acupotomy + PVB CG: PVB	1. Evaluation score (35/35)	6 months
Zhu (2018)^[20]^	TG: 36–62 (49 ± 13) CG: 40–62 (51 ± 11)	TG: 24/26 CG: 23/27	100 (50:50)	CSR	TG: 1.3 ± 0.6 years CG: 1.2 ± 0.7 years	TG: Acupotomy + SNRB CG: SNRB	1. Pain VAS 2. TER (%)	3 months
Pu (2023)^[21]^	TG: 55.6 ± 10.5 CG: 52.5 ± 11.5	TG: 42/38 CG: 41/39	160 (80:80)	CSR	TG: 2.0 weeks CG: 2.0 weeks	TG: Acupotomy + SNRB CG: SNRB	1. Excellent and good rate (%) 2. TER (%) 3. Pain VAS 4. NDI	6 months

CG = control group, CSR = cervical spondylotic radiculopathy, F/U = follow up, N.R. = not reported, NDI = neck disability index, PVB = paravertebral block, SNRB = selective nerve root block, TER = total effective rate, TG = treatment group, VAS = visual analog score.

### 3.3. Analysis of the treatment and control groups

#### 3.3.1. Acupotomy

Three studies specified the types or shapes of needles used in their acupotomy treatments. However, only one study mentioned the thickness and length of the needle used. In 3 of the 4 studies, the acupotomy procedure was performed once a week for a total of 3 to 4 sessions, whereas in Pu et al^[21]^’s study, only 1 to 2 sessions were conducted. Three studies relied on the help of a guiding tool for performing acupotomy, such as a C-arm or ultrasound. Zhu et al^[20]^ and Lu et al^[19]^ utilized C-arm fluoroscopy; Pu et al^[21]^ used ultrasound guidance using the Voluson P8 (General Electric, Boston, Massachusetts.) equipped with a 5 to 12 MHz high-frequency linear array probe (Table S2, Supplemental Digital Content, https://links.lww.com/MD/P143).

#### 3.3.2. Nerve block therapies

All 4 studies specified the types or shapes of NBT administered to their populations, where 0.7-mm needles were used. In 3 studies, the chosen anesthetic was lidocaine, while betamethasone was the steroid used in 2 studies. The frequency and number of sessions were the same as those of the acupotomy procedure (Table S2, Supplemental Digital Content, https://links.lww.com/MD/P143).

### 3.4. Assessing the risk of bias

**Figure 2.**
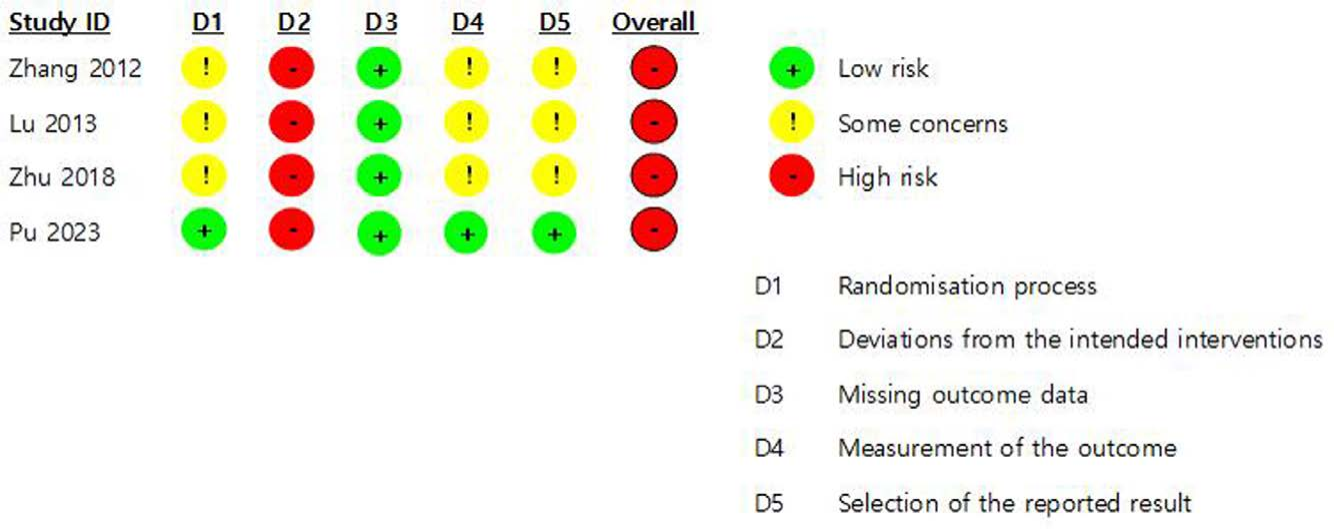
Risk of bias summary.

We concluded that the majority of the studies presented some concerns as they did not describe their random selection processes in detail. The study by Pu et al^[21]^ used random number tables to assign patients and was deemed to have a low risk of bias. As for standard deviations from the intended interventions, all 4 studies were deemed to carry high risks of bias, as they involved manifestly different interventions for the treatment and control groups while avoiding blinding. The risk of bias due to missing outcome data was assessed to be low for all 4 studies, as none reported missing outcomes. The majority of the studies presented concerns with respect to outcomes, as they made no mention of blinding evaluators and had their patients directly assess the effectiveness of their treatments. The only study that explicitly mentioned blinding evaluators, Pu et al^[21]^ was assessed as having a low risk of bias in this regard. As most studies lacked reports or confirmations of protocol studies, we could not assess the risk of publication bias, other than concluding that they presented some concerns. However, the study by Pu et al^[21]^ which was preceded by a protocol study to validate its research design, was assessed to carry a low risk of publication bias. Despite these variations, the overall risk of bias was high for all 4 studies (Fig. 2).

### 3.5. Meta-analysis

#### 3.5.1. Pain VAS

**Figure 3.**
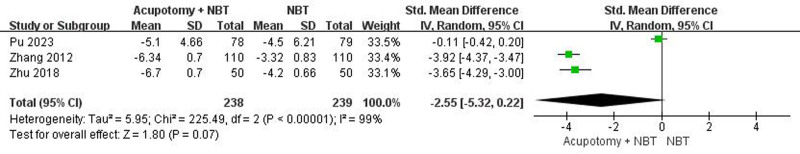
Meta-analysis of the pain visual analog scale.

We subjected 3 studies using the pain VAS to a meta-analysis. We found a significant level of heterogeneity between the studies (I² = 99%); thus, we used a random effects model. Our meta-analysis revealed that patients in the treatment groups were not statistically significantly lower in pain VAS than patients in the control groups (SMD ‐2.55; 95% CI ‐5.32 to ‐0.22; *P* = .07) (Fig. 3).

#### 3.5.2. Total effective rate

**Figure 4.**
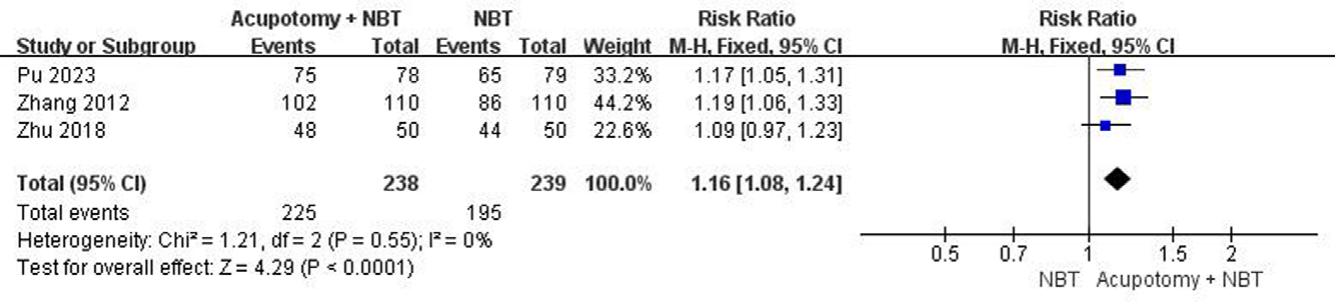
Meta-analysis of the total effective rate.

The 3 studies using TER were subjected to a meta-analysis. With the heterogeneity among them estimated at I² = 0%, we employed a fixed-effects model. Our meta-analysis revealed that the TER, on average, was statistically significantly higher in the treatment group than in the control group (risk ratio 1.16; 95% CI 1.08–1.24; *P* < .0001) (Fig. 4).

### 3.6. Adverse events

One study mentioned AEs. Pu et al^[21]^ reported 2 cases of anomalous blood vessel vagus nerve responses in the treatment group and one in the control group during treatment, all of which disappeared after symptomatic treatment.

### 3.7. Sensitivity analysis

We performed a sensitivity analysis in relation to the pain VAS and TER to evaluate the robustness of the results. Regarding the pain VAS, removing the study by Pu et al^[21]^ resulted in more consistent results with lower heterogeneity, suggesting that this study was a major cause of high heterogeneity and had a substantial influence on the overall result. In contrast, excluding individual studies from the TER analysis had no impact on the overall effect size, indicating the robustness of the result (Table S3, Supplemental Digital Content, https://links.lww.com/MD/P144).

### 3.8. Assessing publication bias

Since this study included fewer than 10 studies, statistical tests for publication bias were not performed, and funnel plots were not created. This limitation is explicitly addressed in the discussion section.

## 4. Discussion

Since acupotomy can effectively address biomechanical factors and NBT can regulate biochemical factors, we expected that combining these interventions could amplify their benefits while minimizing their limitations. Therefore, we conducted a SR of the literature on acupotomy and NBT as treatments for CR, accompanied by a meta-analysis, following our preestablished protocols to assess the effectiveness and safety of their combination.

In this study, the 4 included studies assessed pain reduction using the pain VAS, treatment effectiveness using the TER, and functional improvement using the NDI. The acupotomy-NBT combination showed higher treatment effectiveness compared to NBT alone. However, the pain reduction did not show a significant effect. Due to the limited number of RCTs assessing NDI, a meta-analysis was not feasible. In terms of safety outcome, only one of the studies made safety-related reports. The lack of safety data in most studies is a major limitation, though the reported AEs were mild and resolved with symptomatic treatment.

The heterogeneity analysis revealed a high level of heterogeneity for the pain VAS (I² = 99%). Sensitivity analysis identified Pu et al^[21]^ as the main source of heterogeneity. This study included only patients with a disease duration of <12 weeks, resulting in a significantly shorter average disease course (2 weeks) compared to other studies. Additionally, while other studies administered the interventions at least 3 times, the study by Pu et al^[21]^ applied the interventions only once, with a second session performed only if no improvement was observed, resulting in a total of only 1 to 2 sessions. Considering that CR is typically caused by degenerative changes in the spine, it is unlikely that 1 to 2 sessions alone would be sufficient to relieve pain in patients with a short period of illness.^[22]^

Previous meta-analyses have focused on either acupotomy or NBT independently,^[23–25]^ highlighting the need for a more comprehensive review based on integrative medicine.^[26]^ No SR has been performed to evaluate the effectiveness of combining the 2 interventions. In response to this gap, our study aimed to bridge Eastern and Western medical interventions and assess their combined effectiveness.

This study is significant for following reasons: First, it is the first meta-analysis evaluating the effectiveness of acupotomy combined with NBT for CR compared to NBT alone. Second, this study is valuable for its use of diverse databases in English, Chinese, and Korean with the aim of finding generalizable results. To reduce the risk of selection bias and enhance the comprehensiveness of our SR, we included studies published in English, Chinese, and Korean. This selection was based on the significant presence of clinical research on acupotomy and NBT within these linguistic and regional domains. Notably, China and Korea are major contributors to research on East Asian medical interventions such as acupotomy, while English remains the predominant language for global clinical publications. Third, it bridges modern Western and Korean medicine in response to a growing trend in recent clinical practice. Integrative medicine has garnered increasing attention across multiple fields of clinical practice over the last few decades,^[27]^ particularly in relation to the treatment of musculoskeletal disorders.^[28]^ This study affirms the effectiveness of integrating Western medicine (NBT) and Korean medicine (acupotomy) in treating CR, a rather prevalent disease, and provides additional evidence supporting the use of integrative medicine for musculoskeletal diseases generally.

This study has several limitations that should be considered when interpreting the results. First, the risk of bias was generally high or unclear across most of the included studies. Many studies did not clearly describe their randomization processes or implement double-blinding, which may have affected the reliability of the findings. Second, all included studies were conducted in China, raising concerns about the generalizability of the results to other populations with different medical practices and healthcare systems. Third, there was a lack of safety data, as only one study reported AEs. This limits our ability to assess the safety profile of the combined acupotomy and NBT approach. Lastly, due to the small number of included studies (n < 10), statistical assessments for publication bias, such as funnel plot analysis, could not be conducted. This raises the possibility of selective publication of positive results.

To address these limitations, future research should include well-designed, multicenter, large-scale RCTs with rigorous methodological quality. Additionally, studies should provide standardized reporting on treatment protocols and evaluate both the short-term and long-term effects of acupotomy combined with NBT. Such efforts would improve the credibility and applicability of the evidence regarding the acupotomy-NBT combination for managing CR.

## 5. Conclusion

This systematic review and meta-analysis suggest that combining acupotomy with NBT may enhance treatment effectiveness for CR, particularly in terms of TER. However, no significant improvement was observed in pain reduction, and the included studies had high risks of bias, substantial heterogeneity, and limited safety reporting. Given these limitations, further well-designed, large-scale RCTs with standardized methodologies and long-term follow-ups are needed. While the acupotomy-NBT combination shows potential as an integrative treatment for CR, more rigorous evidence is required to confirm its effectiveness and safety.

## Author contributions

**Conceptualization:** Jin-Hyun Lee, Tae-Yong Park.

**Data curation:** Hyeon-Kyu Choi, Sang-Hyun Lee.

**Formal analysis:** Hyeon-Kyu Choi, Sang-Hyun Lee.

**Funding acquisition:** Tae-Yong Park.

**Investigation:** Sang-Hyun Lee.

**Methodology:** Jin-Hyun Lee.

**Project administration:** Young Il Kim, Tae-Yong Park.

**Resources:** Sooil Choi, Sukhee Park.

**Software:** Hyeon-Kyu Choi, Sang-Hyun Lee.

**Supervision:** Young Su Lim, Young Il Kim, Tae-Yong Park.

**Validation:** Jin-Hyun Lee, Young Su Lim.

**Visualization:** Hye-Jung Kim.

**Writing – original draft:** Hyeon-Kyu Choi.

**Writing – review & editing:** Sang-Hyun Lee, Jin-Hyun Lee.

## Supplementary Material


