# Supplementary material for: Effectiveness of acupotomy combined with nerve block therapy for cervical radiculopathy: A systematic review and meta-analysis
Source: Medicine (Baltimore). 2025 Jun 13;104(24):e42771. doi: 10.1097/MD.0000000000042771 (PMC12173307; doi:10.1097/MD.0000000000042771)
Supplement: Supplementary file 1 [file medi-104-e42771-s001.docx]

| **Table S1. Search methods** | | |
| --- | --- | --- |
| **PubMed** | | |
| #1 | acupotom*[tiab] | 202 |
| #2 | acupotomology[tiab] | 19 |
| #3 | needle knife[tiab] | 843 |
| #4 | needle scalpel[tiab] | 16 |
| #5 | miniscalpel[tiab] | 20 |
| #6 | stiletto needle[tiab] | 9 |
| #7 | sword like needle[tiab] | 1 |
| #8 | mini needle knife[tiab] | 3 |
| #9 | xiaozhendao[tiab] | 1 |
| #10 | #1 or #2 or #3 or #4 or #5 or #6 or #7 or #8 or #9 | 1,055 |
| #11 | nerve block[mh] | 27,484 |
| #12 | nerve block*[tiab] | 17,506 |
| #13 | chemical neurolys*[tiab] | 107 |
| #14 | chemodenervation*[tiab] | 508 |
| #15 | local anesthetic nerve block*[tiab] | 35 |
| #16 | regional nerve block*[tiab] | 474 |
| #17 | conduction block[tiab] | 3,994 |
| #18 | nerve block anaesthesia[tiab] | 56 |
| #19 | neurogenic blockade[tiab] | 10 |
| #20 | epidural steroid injection*[tiab] | 1,905 |
| #21 | epidural administration[tiab] | 1,002 |
| #22 | epidural application[tiab] | 103 |
| #23 | epidural injection*[tiab] | 2,301 |
| #24 | epidural treatment[tiab] | 44 |
| #25 | epidural block[tiab] | 1,664 |
| #26 | medial branch block[tiab] | 156 |
| #27 | selective nerve root block[tiab] | 152 |
| #28 | paravertebral block[tiab] | 1,258 |
| #29 | nerve root block[tiab] | 357 |
| #30 | epidural anesthesia[mh] | 14,380 |
| #31 | #11 or #12 or #13 or #14 or #15 or #16 or #17 or #18 or #19 or #20 or #21 or #22 or #23 or #24 or #25 or #26 or #27 or #28 or #29 or #30 | 57,838 |
| #32 | #10 and #31 | 7 |
| #33 | radiculopathy[mh] | 6,133 |
| #34 | radiculopath*[tiab] | 8,793 |
| #35 | cervical radiculopath*[tiab] | 1,790 |
| #36 | nerve root disorder*[tiab] | 18 |
| #37 | radiculiti*[tiab] | 949 |
| #38 | nerve root inflammation*[tiab] | 26 |
| #39 | nerve root avulsion*[tiab] | 217 |
| #40 | nerve root compression*[tiab] | 1,263 |
| #41 | cervicobrachial neuralgia[tiab] | 82 |
| #42 | #33 or #34 or #35 or #36 or #37 or #38 or #39 or #40 or #41 | 13,440 |
| #43 | #32 and #42 | 3 |
| **Embase** | | |
| #1 | acupotom*:ti,ab,kw | 253 |
| #2 | acupotomology:ti,ab,kw | 29 |
| #3 | ‘needle knife’:ti,ab,kw | 1,843 |
| #4 | ‘needle scalpel’:ti,ab,kw | 33 |
| #5 | miniscalpel:ti,ab,kw | 22 |
| #6 | ‘stiletto needle’:ti,ab,kw | 9 |
| #7 | ‘sword like needle’:ti,ab,kw | 5 |
| #8 | ‘mini needle knife’:ti,ab,kw | 0 |
| #9 | xiaozhendao:ti,ab,kw | 2 |
| #10 | #1 OR #2 OR #3 OR #4 OR #5 OR #6 OR #7 OR #8 OR #9 | 2,118 |
| #11 | ‘nerve block’/exp | 56,907 |
| #12 | ‘nerve block*’:ti,ab,kw | 25,395 |
| #13 | ‘chemical neurolys*’:ti,ab,kw | 177 |
| #14 | chemodenervation*:ti,ab,kw | 767 |
| #15 | ‘local anesthetic nerve block*’:ti,ab,kw | 53 |
| #16 | ‘regional nerve block*’:ti,ab,kw | 673 |
| #17 | ‘conduction block’:ti,ab,kw | 6,224 |
| #18 | ‘nerve block anaesthesia’:ti,ab,kw | 72 |
| #19 | ‘neurogenic blockade’:ti,ab,kw | 16 |
| #20 | ‘epidural steroid injection*’:ti,ab,kw | 2,874 |
| #21 | ‘epidural administration’:ti,ab,kw | 1,283 |
| #22 | ‘epidural application’:ti,ab,kw | 143 |
| #23 | ‘epidural injection*’:ti,ab,kw | 3,141 |
| #24 | ‘epidural treatment’:ti,ab,kw | 58 |
| #25 | ‘epidural block’:ti,ab,kw | 2,412 |
| #26 | ‘medial branch block’:ti,ab,kw | 275 |
| #27 | ‘selective nerve root block’:ti,ab,kw | 201 |
| #28 | ‘paravertebral block’:ti,ab,kw | 1,911 |
| #29 | ‘nerve root block’:ti,ab,kw | 424 |
| #30 | ‘epidural anesthesia’/exp | 37,574 |
| #31 | #11 OR #12 OR #13 OR #14 OR #15 OR #16 OR #17 OR #18 OR #19 OR #20 OR #21 OR #22 OR #23 OR #24 OR #25 OR #26 OR #27 OR #28 OR #29 OR #30 | 104,754 |
| #32 | #10 AND #31 | 13 |
| #33 | radiculopathy/exp | 49,222 |
| #34 | radiculopath*:ti,ab,kw | 12,711 |
| #35 | ‘cervical radiculopath*’:ti,ab,kw | 2,486 |
| #36 | ‘nerve root disorder*’:ti,ab,kw | 29 |
| #37 | radiculiti*:ti,ab,kw | 1,275 |
| #38 | ‘nerve root inflammation*’:ti,ab,kw | 41 |
| #39 | ‘nerve root avulsion*’:ti,ab,kw | 276 |
| #40 | ‘nerve root compression*’:ti,ab,kw | 1,675 |
| #41 | ‘cervicobrachial neuralgia’/exp | 4,423 |
| #42 | #33 OR #34 OR #35 OR #36 OR #37 OR #38 OR #39 OR #40 OR #41 | 56,632 |
| #43 | #32 AND #42 | 4 |
| **Ovid** | | |
| 1 | acupotom*.mp. | 202 |
| 2 | acupotomology.mp. | 19 |
| 3 | needle knife.mp. | 845 |
| 4 | needle scalpel.mp. | 16 |
| 5 | miniscalpel.mp. | 20 |
| 6 | stiletto needle.mp. | 9 |
| 7 | sword like needle.mp. | 5 |
| 8 | mini needle knife.mp. | 0 |
| 9 | xiaozhendao.mp. | 1 |
| 10 | or/1-9 | 1,060 |
| 11 | exp nerve block/ | 27,506 |
| 12 | nerve block*.mp. | 34,547 |
| 13 | chemical neurolys*.mp. | 107 |
| 14 | chemodenervation*.mp. | 491 |
| 15 | local anesthetic nerve block*.mp. | 35 |
| 16 | regional nerve block*.mp. | 486 |
| 17 | conduction block.mp. | 3,993 |
| 18 | nerve block anaesthesia.mp. | 57 |
| 19 | neurogenic blockade.mp. | 10 |
| 20 | epidural steroid injection*.mp. | 1,908 |
| 21 | epidural administration.mp. | 1,003 |
| 22 | epidural application.mp. | 103 |
| 23 | epidural injection*.mp. | 2,301 |
| 24 | epidural treatment.mp. | 45 |
| 25 | epidural block.mp. | 1,664 |
| 26 | medial branch block.mp. | 156 |
| 27 | selective nerve root block.mp. | 152 |
| 28 | paravertebral block.mp. | 1,262 |
| 29 | nerve root block.mp. | 327 |
| 30 | exp epidural anesthesia/ | 14,387 |
| 31 | or/11-30 | 57,877 |
| 32 | 10 and 31 | 7 |
| 33 | exp radiculopathy/ | 6,37 |
| 34 | radiculopath*.mp. | 11,943 |
| 35 | cervical radiculopath*.mp. | 1,791 |
| 36 | nerve root disorder*.mp. | 18 |
| 37 | radiculiti*.mp. | 948 |
| 38 | nerve root inflammation*.mp. | 26 |
| 39 | nerve root avulsion*.mp. | 217 |
| 40 | nerve root compression*.mp. | 1,261 |
| 41 | cervicobrachial neuralgia.mp. | 82 |
| 42 | or/33-41 | 13,446 |
| 43 | 32 and 42 | 3 |
| **CENTRAL** | | |
| #1 | acupotom* | 164 |
| #2 | acupotomology | 14 |
| #3 | “needle knife” | 237 |
| #4 | “needle scalpel” | 10 |
| #5 | miniscalpel | 20 |
| #6 | “stiletto needle” | 6 |
| #7 | “sword like needle” | 1 |
| #8 | “mini needle knife” | 1 |
| #9 | xiaozhendao | 0 |
| #10 | #1 or #2 or #3 or #4 or #5 or #6 or #7 or #8 or #9 | 420 |
| #11 | MeSH descriptor: [Nerve Block] explode all trees | 6,058 |
| #12 | “nerve block” | 14,920 |
| #13 | “chemical neurolysis” | 29 |
| #14 | chemodenervation | 51 |
| #15 | “local anesthetic nerve block” | 18 |
| #16 | “regional nerve block” | 137 |
| #17 | “conduction block” | 241 |
| #18 | “nerve block anaesthesia” | 209 |
| #19 | “neurogenic blockade” | 5 |
| #20 | “epidural steroid injection” | 406 |
| #21 | “epidural administration” | 436 |
| #22 | “epidural application” | 34 |
| #23 | “epidural injection” | 788 |
| #24 | “epidural treatment” | 18 |
| #25 | “epidural block” | 1,179 |
| #26 | “medial branch block” | 83 |
| #27 | “selective nerve root block” | 46 |
| #28 | “paravertebral block” | 1,434 |
| #29 | “nerve root block” | 110 |
| #30 | MeSH descriptor: [Anestehsia, Epidural] explode all trees | 2,340 |
| #31 | #11 or #12 or #13 or #14 or #15 or #16 or #17 or #18 or #19 or #20 or #21 or #22 or #23 or #24 or #25 or #26 or #27 or #28 or #29 or #30 | 20,415 |
| #32 | #10 and #31 | 19 |
| #33 | MeSH descriptor: [Radiculopathy] explode all trees | 781 |
| #34 | radiculopathy | 2,251 |
| #35 | “cervical radiculopathy” | 547 |
| #36 | “nerve root disorder” | 1 |
| #37 | radiculitis | 91 |
| #38 | “nerve root inflammation” | 8 |
| #39 | “nerve root avulsion” | 3 |
| #40 | “nerve root compression” | 219 |
| #41 | “cervicobrachial neuralgia” | 239 |
| #42 | radiculitides | 1 |
| #43 | “cervical radiculopathies” | 9 |
| #44 | #33 or #34 or #35 or #36 or #37 or #38 or #39 or #40 or #41 or #42 or #43 | 2,513 |
| #45 | #32 and #44 | 8 |
| **CNKI** | | |
| 1 | (SU=(‘针刀’ OR ‘针刀松解术’ OR ‘小针刀’ OR ‘小针刀疗法’ OR ‘acupotomy’ OR ‘miniscalpel’) OR TI=(‘针刀’ OR ‘针刀松解术’ OR ‘小针刀’ OR ‘小针刀疗法’ OR ‘acupotomy’ OR ‘miniscalpel’) OR AB=(‘针刀’ OR ‘针刀松解术’ OR ‘小针刀’ OR ‘小针刀疗法’ OR ‘acupotomy’ OR ‘miniscalpel’) OR KY=(‘针刀’ OR ‘针刀松解术’ OR ‘小针刀’ OR ‘小针刀疗法’ OR ‘acupotomy’ OR ‘miniscalpel’)) AND ((SU=(‘神经根型颈椎病’ OR ‘颈椎神经根病’ OR ‘颈神经根性脊椎病’ OR ‘颈椎间盘突出症’ OR ‘颈椎病’ OR ‘颈椎管狭窄’ OR ‘Cervical Radiculopathy’) OR TI=(‘神经根型颈椎病’ OR ‘颈椎神经根病’ OR ‘颈神经根性脊椎病’ OR ‘颈椎间盘突出症’ OR ‘颈椎病’ OR ‘颈椎管狭窄’ OR ‘Cervical Radiculopathy’) OR AB=(‘神经根型颈椎病’ OR ‘颈椎神经根病’ OR ‘颈神经根性脊椎病’ OR ‘颈椎间盘突出症’ OR ‘颈椎病’ OR ‘颈椎管狭窄’ OR ‘Cervical Radiculopathy’) OR KY=(‘神经根型颈椎病’ OR ‘颈椎神经根病’ OR ‘颈神经根性脊椎病’ OR ‘颈椎间盘突出症’ OR ‘颈椎病’ OR ‘颈椎管狭窄’ OR ‘Cervical Radiculopathy’)) AND (SU=(‘神经阻滞’ OR ‘神经传导阻滞’ OR ‘nerve block’ OR ‘硬膜外腔激素注射’ OR ‘硬膜外类固醇注射’ OR ‘硬膜外类固醇注射疗法’ OR ‘epidural steroid injection’ OR ‘内侧支传导阻滞’ OR ‘medial branch block’ OR ‘选择性神经根阻滞’ OR ‘selective nerve root block’) OR TI=(‘神经阻滞’ OR ‘神经传导阻滞’ OR ‘nerve block’ OR ‘硬膜外腔激素注射’ OR ‘硬膜外类固醇注射’ OR ‘硬膜外类固醇注射疗法’ OR ‘epidural steroid injection’ OR ‘内侧支传导阻滞’ OR ‘medial branch block’ OR ‘选择性神经根阻滞’ OR ‘selective nerve root block’) OR AB=(‘神经阻滞’ OR ‘神经传导阻滞’ OR ‘nerve block’ OR ‘硬膜外腔激素注射’ OR ‘硬膜外类固醇注射’ OR ‘硬膜外类固醇注射疗法’ OR ‘epidural steroid injection’ OR ‘内侧支传导阻滞’ OR ‘medial branch block’ OR ‘选择性神经根阻滞’ OR ‘selective nerve root block’) OR KY=(‘神经阻滞’ OR ‘神经传导阻滞’ OR ‘nerve block’ OR ‘硬膜外腔激素注射’ OR ‘硬膜外类固醇注射’ OR ‘硬膜外类固醇注射疗法’ OR ‘epidural steroid injection’ OR ‘内侧支传导阻滞’ OR ‘medial branch block’ OR ‘选择性神经根阻滞’ OR ‘selective nerve root block’)) | 85 |
| **OASIS** | | |
| 1 | 도침 경추 신경차단술 | 0 |
| 2 | 도침 경추 경막외스테로이드 | 0 |
| 3 | 도침 경추 내측 분지 차단술 | 0 |
| 4 | 도침 경추 선택적 신경근 차단술 | 0 |
| 5 | 침도 경추 신경차단술 | 0 |
| 6 | 침도 경추 경막외스테로이드 | 0 |
| 7 | 침도 경추 내측 분지 차단술 | 0 |
| 8 | 침도 경추 선택적 신경근 차단술 | 0 |
| **KCI** | | |
| 1 | 도침 AND 경추 AND 신경차단술 | 0 |
| 2 | 도침 AND 경추 AND 경막외스테로이드 | 0 |
| 3 | 도침 AND 경추 AND 내측 분지 차단술 | 0 |
| 4 | 도침 AND 경추 AND 선택적 신경근 차단술 | 0 |
| 5 | 침도 AND 경추 AND 신경차단술 | 0 |
| 6 | 침도 AND 경추 AND 경막외스테로이드 | 0 |
| 7 | 침도 AND 경추 AND 내측 분지 차단술 | 0 |
| 8 | 침도 AND 경추 AND 선택적 신경근 차단술 | 0 |
| **RISS** | | |
| 1 | 전체 : 도침 <AND> 전체 : 경추 <AND> 전체 : 신경차단술 | 0 |
| 2 | 전체 : 도침 <AND> 전체 : 경추 <AND> 전체 : 경막외스테로이드 | 0 |
| 3 | 전체 : 도침 <AND> 전체 : 경추 <AND> 전체 : 내측 분지 차단술 | 0 |
| 4 | 전체 : 도침 <AND> 전체 : 경추 <AND> 전체 : 선택적 신경근 차단술 | 0 |
| 5 | 전체 : 침도 <AND> 전체 : 경추 <AND> 전체 : 신경차단술 | 0 |
| 6 | 전체 : 침도 <AND> 전체 : 경추 <AND> 전체 : 경막외스테로이드 | 1 |
| 7 | 전체 : 침도 <AND> 전체 : 경추 <AND> 전체 : 내측 분지 차단술 | 0 |
| 8 | 전체 : 침도 <AND> 전체 : 경추 <AND> 전체 : 선택적 신경근 차단술 | 0 |
| **KISS** | | |
| 1 | 전체 = “도침” and 전체 = “경추” and 전체 = “신경차단술” | 0 |
| 2 | 전체 = “도침” and 전체 = “경추” and 전체 = “경막외스테로이드” | 0 |
| 3 | 전체 = “도침” and 전체 = “경추” and 전체 = “내측 분지 차단술” | 0 |
| 4 | 전체 = “도침” and 전체 = “경추” and 전체 = “선택적 신경근 차단술” | 0 |
| 5 | 전체 = “침도” and 전체 = “경추” and 전체 = “신경차단술” | 0 |
| 6 | 전체 = “침도” and 전체 = “경추” and 전체 = “경막외스테로이드” | 0 |
| 7 | 전체 = “침도” and 전체 = “경추” and 전체 = “내측 분지 차단술” | 0 |
| 8 | 전체 = “침도” and 전체 = “경추” and 전체 = “선택적 신경근 차단술” | 0 |
| **ScienceON** | | |
| 1 | 전체=(도침\|침도) AND 전체=경추 AND 전체=(신경차단술\|경막외스테로이드\|내측 분지 차단술\|선택적 신경근 차단술) | 0 |
