# Supplementary material for: Effectiveness of acupotomy combined with nerve block therapy for cervical radiculopathy: A systematic review and meta-analysis
Source: Medicine (Baltimore). 2025 Jun 13;104(24):e42771. doi: 10.1097/MD.0000000000042771 (PMC12173307; doi:10.1097/MD.0000000000042771)
Supplement: Supplementary file 2 [file medi-104-e42771-s002.docx]

| Table S2. Intervention methods | | | | | | | | |
| --- | --- | --- | --- | --- | --- | --- | --- | --- |
| Author (Year) | **Acupotomy** | | | **NBT** | | | | **Guide Tools** |
|  | **Needle Insertion Sites** | **Needle Types** | **Frequency and Number** | **Injected Solutions** | **Needle Insertion Sites** | **Needle Types** | **Frequency and Number** |  |
| Zhang (2012) | Between the affected side spinous process and transverse process | N.R. | 1 time/week,  4 times | 2% lidocaine 5 ml, bupivacaine 3 ml, Danhong injection 5–8 ml, adenosine cobalamin 3 mg, citicoline 500 mg, prednisolone 10–20 mg, total dose 20 ml | 2 cm next to the point between the spinous process and the transverse process | No.7 10 ml puncture needle | 1 time/week,  4 times | N.R. |
| Lu (2013) | Transverse process nodules of C3–C7 nerve root lesions (<4) | Hanzhang III needle type acupuncture needle | 1 time/week,  3 times | 2% lidocaine injection solution 2.5 ml, triamcinolone acetonide injection 10 mg, methylcobalamin injection 500 µ g, normal saline 5 ml | Transverse process nodules of C3–C7 nerve root lesions | No.7 10 ml puncture needle | 1 time/week,  3 times | C-arm fluoroscopy |
| Zhu (2018) | Adhesion of the posterior tubercle and intertubercular sulcus of the cervical spine | Hanzhang III/IV Needle Knife | 1 time/week,  3 times | 2% lidocaine hydrochloride 2.5 ml, compound betamethasone 1 ml, 0.9% sodium chloride solution 20 ml | Posterior tubercle | No.7 puncture needle | 1 time/week,  3 times | C-arm fluoroscopy |
| Pu (2023) | Posterior tubercle of C5–7 transverse process | Hanqing disposable sterile injection needle knife 0.9*85mm (Henan Shangrui Med Tech Co., Ltd., Henan, China) | 1–2 times (second treatment was given one week later) | betamethasone injection (Schering-Plough Labo N.V., Berg, Belgium), 1.5 ml of 2% lidocaine hydrochloride (Shanxi Jinxin Double Crane Pharmaceutical Co., Ltd., Shanxi, China), and 3.5 ml of 0.9% sodium chloride (Sinopharm Rongsheng Pharmaceutical Co., Ltd., Henan, China), total 6 ml | Near the cervical nerve root between the anterior and posterior nodules | 10 ml, 22 G 0.7*80 mm ultrasound-guided special nerve block needle (Shenzhen Tuoren Biomedical Electronics Co., Ltd., Guangdong, China) | 1–2 times (second treatment was given one week later) | Ultrasound positioning |
| NBT: Nerve block therapy, N.R.: Not reported | | | | | | | | |
