# Supplementary material for: Effectiveness of acupotomy combined with nerve block therapy for cervical radiculopathy: A systematic review and meta-analysis
Source: Medicine (Baltimore). 2025 Jun 13;104(24):e42771. doi: 10.1097/MD.0000000000042771 (PMC12173307; doi:10.1097/MD.0000000000042771)
Supplement: Supplementary file 3 [file medi-104-e42771-s003.docx]

| **Table S3. Sensitivity analysis** | | | | | |
| --- | --- | --- | --- | --- | --- |
| **Study omitted** | **Pooled estimate** | **95% Confidence interval** | | **p-value** | ***I^2^*(%)** |
|  |  | **Lower** | **Upper** |  |  |
| Pain VAS | | | | | |
| Pu 2023 | -3.83 | -4.20 | -3.46 | <0.001 | 0 |
| Zhang 2012 | -1.87 | -5.33 | 1.60 | 0.29 | 99 |
| Zhu 2018 | -2.01 | -5.75 | 1.72 | 0.29 | 99 |
| TER | | | | | |
| Pu 2023 | 1.15 | 1.06 | 1.26 | 0.0009 | 10 |
| Zhang 2012 | 1.14 | 1.05 | 1.23 | 0.002 | 0 |
| Zhu 2018 | 1.18 | 1.09 | 1.28 | <0.001 | 0 |
| pain VAS: pain Visual Analogue Scale score, TER: total effective rate | | | | | |
